# Unbalanced Flows in the Subtle Body: Tibetan Understandings of Psychiatric Illness and How to Deal With It

**DOI:** 10.1007/s10943-019-00774-1

**Published:** 2019-02-20

**Authors:** Geoffrey Samuel

**Affiliations:** 1 School of History, Archaeology and Religion, Cardiff University, Cardiff, UK; 2 School of Languages and Cultures, University of Sydney, Sydney, Australia

**Keywords:** Psychiatric illness, Tibet, Buddhism, Tantra, Subtle body, Autonomic nervous system

## Abstract

Much of what Western medicine classifies as psychiatric illness is understood by Tibetan thought as associated with imbalance of *rlung* (wind, breath). *Rlung* has a dual origin in Indian thought, combining elements from Ayurvedic medicine and Tantric Buddhism. Tibetan theories of *rlung* seem to correspond in significant ways with Western concepts of the autonomic nervous system (ANS), and Western medicine too has associated psychiatric issues with ANS problems. But what is involved in relating Tibetan ideas of *rlung* to Western ideas of the emotions and the ANS? The article presents elements of the two systems and then explores similarities and differences between them. It asks whether the similarities could be the basis for a productive encounter between Tibetan and Western modes of understanding and treating psychiatric illness. What could Western psychiatry learn from Tibetan approaches in this area?

## Introduction

As Susannah Deane’s article in this collection explains (Deane, this issue), much of the domain of psychiatric illness in biomedicine is understood in Tibetan medicine in terms of disorders in an internal process known as *rlung* (pronounced ‘loong’), sometimes translated as ‘wind.’ In this article, I examine the use of *rlung* as an explanation for psychiatric illness and ask how it might relate to biomedical and neuroscientific modes of explanation for psychiatric disorders.

My argument proceeds in several stages. I begin by discussing the term *rlung* and its origins. I proceed to look at how it led to a series of categories which Tibetans used to understand some of the area we would describe as psychiatric illness. I then turn to consider *rlung* in the Tantric context and discuss possible relationships between the subtle anatomy of Tantric yoga and Western concepts of the autonomic nervous system. Finally, I look at psychiatric illness in terms of states of imbalance in the subtle body and in terms of disorders in the autonomic nervous system. Can we construct a meaningful relationship between the two, and what light might this throw on each?

## *Rlung* and Its Meanings in Tibet

*Rlung* is one of three pathogenic processes (*nyes pa*), *rlung, mkhris pa* and *bad kan,* which are central to Tibetan medical theory. The names for the three *nyes pa* are translations of three parallel Sanskrit terms found in Ayurvedic medicine, from which Tibetan medicine derives much of its theoretical structure. These three Sanskrit terms (*vāyu* or *vāta*, *pitta* and *kapha*) are known as the three *doṣa*.

Ideas concerning *rlung* coexist in Tibetan psychiatric theory with concepts of *karma*, ideas about the action of spirits and deities, as well as with Buddhist philosophical concepts, but unbalanced or disordered flows of *rlung* are nevertheless a key way in which psychiatric disorders are explained. While *rlung* is often translated as ‘wind,’ this is as partial and misleading a translation as ‘bile’ and ‘phlegm’ for the other two *nyes pa,* or for the Ayurvedic *doṣa* from which they derive. What is at issue is not ‘wind,’ ‘bile’ or ‘phlegm’ in the literal sense, but an internal process encompassing both mind and body for which ‘wind’ (or rather *rlung*) serves as a conventional label.^1^ While the *nyes pa* are sometimes referred to as ‘humours,’ this term, as Deane notes, is best avoided (cf also Y. Gyatso 2005–2006). These are not factors which should be kept in balance with each other, as with the ‘humours’ of the pre-modern European medical tradition, but processes within the body—mind system any of which can become excessive and so damaging to the body—mind as a whole.

As Deane also mentioned in her article, Tibetan medical theory—here differing from Ayurveda—sees these three processes of ‘wind,’ ‘bile’ and ‘phlegm’ as rooted in three more fundamental processes that are part of Buddhist rather than medical theory. These are the Three Poisons (*dug gsum*) or three basic obscurations or afflictive mental factors (*nyon mongs gsum*): attachment or greed, aversion or anger, and delusion or confusion (e.g. Wayman 1957). *Nyon mongs* translates Sanskrit *kleśa*, often rendered in English as ‘obscuration’ or ‘defilement,’ sometimes as ‘destructive emotion’ or ‘afflictive mental factor.’^2^ In Pali Buddhism, attachment, aversion and delusion are referred to as the ‘three unwholesome roots.’ For all Buddhists, these are the three key factors that drive the everyday world of *saṃsāra* from which Buddhism offers liberation. From that point of view, they underlie illness, as they underlie all the other evils and sufferings of *saṃsāra*. The Tibetan medical classic, the *Rgyud bzhi* or ‘Four Medical Tantras,’ now generally accepted to be the work of g.Yu thog Yon tan mgon po in the late twelfth century (Ga 2010), however, goes further than this. It describes the three pathogenic processes of *rlung, mkhris pa* and *bad kan* as arising, respectively, from the Three Poisons of attachment, aversion and delusion, which in turn are rooted in the ignorance of our true nature, which for Mahāyāna Buddhists like the Tibetans is the ultimate cause of the Three Poisons themselves.^3^ This gives a specifically psychological dimension to illness which is not present in the same way in the Ayurvedic medicine of South Asia (cf Ga 2010: 162).

What this means for the practice of Tibetan medicine is a complex question, and Tibetan practitioners vary in how far they engage with this psychological or spiritual level of explanation. Minimally, though, it builds a much stronger and more direct role for mind, consciousness and what we might label as emotional states into Tibetan medicine than can be found within Ayurveda, or arguably within mainstream Western medicine. In relation to *rlung* specifically, the connection with desire or attachment is frequently made. Craig Janes, an American medical anthropologist who carried out research in Tibetan medical practice in Central Tibet, the Tibet Autonomous Region of the Chinese People’s Republic, in the late 1980s and early 1990s, comments that.

In Buddhist medical theory, *rlung* imbalance is linked most closely to the mental poison of desire (*’dod chags*). When asked to discuss the relationship of desire to wind imbalance, the physicians I interviewed tended to provide operational definitions that highlighted the disjuncture between socially legitimate hopes and actualities. For example, one physician described how desire manifests itself among average people and how it may lead to an imbalance of *rlung*: “People want to have good living conditions, enough food, obedient children, peace in the family, and so on. However, when they do not have these things that they desire, it leads to mental agitations, and these in turn cause *rlung* imbalance.” (Janes 1995: 30).

The idea that one can have excessive *rlung*, high *rlung*, too much *rlung*, is very common among Tibetans (Jacobson 2007: 232). Since *rlung* is, as we shall see, closely linked to consciousness, this carries implications of too much thinking, becoming too carried away by emotions, and the like.

*Rlung,* translating *vāta* or *vāyu*, which in Sanskrit are two more or less equivalent terms for ‘wind’ used in the general as well as the medical context, is thus a central term in the Tibetan medical vocabulary. But *rlung* is also a central term in another Tibetan vocabulary, that of Vajrayāna or Buddhist Tantra, which also originates from South Asia (Samuel 2008). Here *rlung* corresponds not to *vāyu* or *vāta,* but a different term, *prāṇa. Prāṇa* is often rendered into English as ‘breath,’ but it refers not just to the process of respiration but to a variety of subtle inner ‘flows’ within the body. Ideas about the flow of *prāṇa* within the body go back to the early Upaniṣads, dating perhaps from the fifth or fourth century BCE. They were developed much further in the context of the growth of Tantric yogic practices in Indian religions from the eighth century CE onwards. In this way, *prāṇa* became an integral part of Tantric ideas regarding the so-called subtle body (Samuel and Johnston 2013), and were transmitted to Tibet along with Buddhist versions of Tantric yoga.

Consequently, Tibetan *rlung* is not the same thing as either Ayurvedic *vāta* or Tantric *prāṇa.* It encompasses elements from both and also has an additional layer of meaning and reference linked to its connection to the fundamental ‘poison’ of attachment or desire. All this is significant in considering how *rlung* comes to be used in relation to psychiatric illness in Tibet.

## *Rlung* and Psychiatric Illness in Tibetan Medicine

The classical work of Tibetan medicine, the *Rgyud bzhi*, the ‘Four (Medical) Tantras,^4^’ discusses *rlung* in many places, since there can be a *rlung* aspect to many disorders. Two particularly important chapters are Chapter 30 of the second Tantra (*Bshad rgyud*), which gives basic treatments for *rlung* disorders, and Chapter 2 of the third Tantra (*Man ngag rgyud*) which gives a general survey of *rlung* disorders. This chapter, the *Rlung gi nad gso ba* or ‘Healing *Rlung* disorders’ chapter, lists twenty individual *rlung* disorders (A warta, two types of Da rgan, ’Gram pa nyams, lCe ldib, etc.), describing the symptoms (*rtags*) and treatment for each (Würthner 2012: 22, 30; see also Jacobson 2000: 172). This list of disorders derives from a variety of sources, some of them Indian, others not (Ga 2010: 183–184). This is followed by various schemes for classifying *rlung* illnesses: six modes by which they enter the body, twenty-eight ways in which they become perceptible to the senses, seven anatomical locations where they may occur (head, heart, lungs, liver, stomach, large intestines, kidneys), and five specific *rlung* that may be affected (Jacobson 2000: 173–176).

Much of this is rarely referred to in contemporary clinical practice. This is true of a considerable part of the material in the *Rgyud bzhi*, which is today often more significant at a conceptual or ideological level than for detailed therapeutic prescriptions. However, elements from some of the schemes in this chapter were developed further in subsequent centuries to provide a series of categories that are widely referred to and used in relation to psychiatric disorders. Here the list of five specific *rlung* was particularly significant: ‘If one divides [*rlung* illnesses] specifically, there are just five: life-sustaining (*srog ’dzin*), rising (*gyen gyu*), pervading (*khyab byed*), fire-accompanying (*me mnyam*) and downwards-driving (*thur sel*).’^5^ These five are the five basic *rlung* (*prāṇa* in Sanskrit) that underlie the operation of the human body (cf. Hartzell 1997: 100–107; Samuel 2008: 284–285). The Sanskrit term here is *prāṇa*, not *vāyu* or *vāta*, but the Tibetan is *rlung,* and these *prāṇa* flows, which come from the late Vedic and yogic rather than the Ayurvedic tradition, are presented by the *Rgyud bzhi* as if they are simply another way of describing the *rlung* flows. The five basic *rlung* were discussed in Deane’s article, but are listed here again for convenience, with the localisation and function of each of the five *rlung* as specified in an earlier chapter of the *Rgyud bzhi*^6^ (translation after Clark 1995: 64) (Table 1):

**Table 1:** The five principal forms of *rlung*

Name	Sanskrit equivalent	Location and movement	Function
Life-sustaining (*srog’dzin*)	*prāṇa*	Located in the crown of the head and travels through the throat and the breastbone	Swallows food and drink, inhales, spits, sneezes, belches, endows the mind and sense organs with clarity and holds the mind [and body together]
Ascending (*gyen gyu*)	*uḍāna*	Located in the chest and runs through the nose, tongue and throat	Projects the speech, provides (physical) strength, complexion, ‘color,’ energy and effort, and clears the memory
Pervading (*khyab byed*)	*vyāna*	Located in the heart and moves throughout the whole body	Raises, presses downwards, moves (the body), stretches, contracts (limbs and digits), opens and closes (the orifices) and is relied upon in the majority of functions
Fire-like equalising (*me mnyam*)	*samāna*	Located in the stomach and runs throughout the internal (vessel) organs	Digests food, separates nutriment and waste products, and nourishes the objects of harm (bodily constituents, excretions etc.)
Downwards voiding (*thur sel*)	*apāna*	Located in the anal area and operates in the large intestine, urinary bladder, genitals and thighs	Discharges and retains the semen, (menstrual) blood, stool, urine and foetus

The *Rgyud bzhi* tells us what the five *rlung* do when operating properly, following closely on the Indian analysis of the five *prāṇa*, and the *Man ngag rgyud*’s chapter on *rlung* illness provides some information about problems associated with their malfunctioning. For disorders in the first three of the five *rlung*, as Eric Jacobson notes, the symptoms are primarily psychiatric in Western terms (Jacobson 2000: 179–180). So are some of the symptoms listed for items in the other classificatory schemes.

In later times, three specific disorders in particular came to be widely recognised. These are listed in Table 2. For the first two I have given summaries of the effects of these disorders from the contemporary doctor Pasang Y. Arya:

**Table 2:** Specific syndromes with psychiatric symptoms

*Srog rlung*	Disorder of life-sustaining (*srog ’dzin*) *rlung* (located in head)	‘One may lose consciousness and balance, manifest vertigo, lose control of the body/mind, have wrong perceptions, confusion, hear sounds in the head and ears, experience feelings of head emptiness, and have hallucinations. It may cause shortness of breath; difficulty of inhalation, problems in swallowing food and drink, and could even become the cause of madness.’ (Arya 2018)
*Snying rlung*	Disorder of pervading (*khyab byed*) *rlung* (located at heart)	‘Manifests in loss of balance, hypertension, chest tension, fear, panic attacks…, fainting, loss of speech, general cardiac disorders, talkativeness, the desire to roam, pains in the joints, shoulders and back, blood circulation disorders, heart palpitations and rhythm disturbances, and in complaining and unfriendly speech which worsens the situation, etc.’ (Arya 2018)
*Khrag rlung*	Involves excess of both *rlung* and *khrag* (‘blood’)	Symptoms of either *snying rlung* or *srog rlung*, along with irritability, headaches, nosebleeds and high blood pressure. (cf Jacobson 2002: 274 n.5)

*Srog rlung*, *snying rlung* and *khrag rlung* became recognised syndromes within Tibetan medicine over the subsequent centuries. Table 3 lists a number of texts from the fifteenth to seventeenth century in which they are discussed. These texts generally give a brief description of the symptoms (*rtags*) of the disorder, followed by a series of recipes for medicines that will treat the disorder.

**Table 3:** Early discussions of srog rlung, snying rlung, and khrag rlung

Author	Work	Chapter or section
Zur mkhar Mnyam nyid rdo je (1439–1475)	*Bye ba ring bsrel*	Two texts on *srog rlung*
dKon mchog phan dar (1511–1577)	*Man ngag yig chung sna tshogs*	Short texts on healing *snying rlung* and *khrag rlung*
Ngag dbang dkon mchog bstan rgyal (1594–1630)	*Man ngag rin chen gter mdzod dang lde mig*	*rlung nad spyi dang srog rlung ce sbyang sogs bcos pa*
Sde srid Sangs rgyas rgya mtsho	*Man ngag lhan thabs*	Sections on *srog rlung* and *khrag rlung*

In the contemporary period, *srog rlung*, *snying rlung* and *khrag rlung* are common diagnostic categories. I shall give a number of examples from different sources.

My first example is of *snying rlung,* from a biography by a contemporary Tibetan lama of one of his relatives, a Tibetan yogic practitioner named Urgyen Tendzin, born in 1888 and living in Eastern Tibet. Ugyen Tendzin went to a local monastery to study at around the age of seven, with the encouragement of his own uncle, who was also a religious practitioner. But the uncle died a few months later, and Ugyen was treated harshly at the monastery. He ran back off home, where his father arranged for him to become a servant for a local aristocratic family to whom he was distantly related. Another servant in the household stole a valuable cup and accused Ugyen of having taken it. Ugyen was struck by a *rlung* disorder. After about a month, the family found out that Ugyen had not taken the bowl, but his disorder continued. After treatment by a Tibetan ritual practitioner, he improved slightly, but some years later, after he had nursed his mother for several months through what proved to be a fatal illness, his illness worsened, and he was confined to a room in the house for some 6 years. Eventually he was taken to a well-known lama, Adzom Drugpa Rinpoche, who gave him further ritual treatment, and took him on as a disciple. (Norbu 2012: 8, 11, 12.)

Urgyen’s disorder is described as *snying rlung*, the form of *rlung* ailment associated with malfunction of the *khyab byed rlung* (Pervasive Wind) which is located in the heart (*snying*) and runs through the internal organs. Norbu’s translator, Adriano Clemente, translates *snying rlung* as ‘wind-in-the-heart illness,’ and characterises it as a kind of depression. He notes that it ‘is generally described as a pathological condition caused by intense suffering and by deterioration of the energy channels of the heart. Its symptoms include remaining blocked or fixated on one thought without being able to think normally’ (Norbu 2012: 88 n.3). While the treatment described in the book is primarily ritual, there are also Tibetan medicinal remedies used in relation to *rlung* disorders, and other therapies (diet, physical exercises) can also help.

A second and more recent example of *snying rlung* is from Craig Janes, whom I have already mentioned. This is from the Tibet Autonomous Region in China, around 1990:

Dawa (age 54) is an important government official in the regional TAR office that sets commodity prices, a controversial office, subject to some loathing on the part of the Tibetan populace. Clearly anxious and agitated when she sees the doctor, Dawa launches immediately into a rapidly spoken litany of complaints, underscored by strong emotion: she is dizzy, her head aches almost constantly, she is frightened by her heart, feeling as if it were swinging wildly in her chest. She has trouble sleeping and when she does, she dreams of her father who died several years ago. Her body, she says, is “unhappy.” Her job is difficult and causes her much worry and anxiety. She is diagnosed with snying-rlung, which the doctor tells her is very serious. She needs to be hospitalized as soon as a bed opens up. The doctor tells her, “As long as you keep working your problem will continue.” (Janes 1999: 397)

Janes implies in his article that Tibetan doctors at this time were systematically using *rlung* diagnoses as a way to relieve the stresses of Chinese occupation, by allowing their patients a substantial period of rest in the relatively protected context of a Tibetan hospital.

A third example, from Eric Jacobson’s fieldwork with Tibetans in the Darjeeling area of West Bengal in the late 1990s, is a case of *khrag rlung. Khrag* means ‘blood’ and can also function as a kind of *nyes pa* in its own right, so *khrag rlung* involves an excess of both blood and *rlung*, in this case *srog rlung*. Kalsang’s illness had begun when her husband’s trading stock had been taken by Chinese soldiers on a trading trip into Tibet. Another Chinese attack led to the loss of most of their remaining resources. The family settled in Darjeeling, but three of their six children died soon after, followed by the husband, and Kalsang was left to bring up her two remaining daughters in a tiny hut among other refugee families. When Jacobson met her, she had been suffering severe *srog rlung* for 2 or 3 years. Her symptoms included anxiety, which affects her breathing, and heart palpitations, along with knee pain and nosebleeds. Jacobson elicited various additional symptoms, which in his view qualified her in terms of DSM-IV for a combination of Generalized Anxiety Disorder and Major Depression^7^:

sadness (at one point she said this lasted two or three days at a time, later “I am sad all the time”), lack of interest in life activities (she was interested only in her housework), insomnia (“When I can’t sleep I remember bad things that happened to my family”), agitation (at the same time as the “electricity” in her legs), difficulty concentrating (sometimes her mind goes completely blank), frequent worry about impending disaster (“I worry that the children may fall down, or fall sick. I worry about that every day. It makes me sick”), dizziness, exaggerated startle responses accompanied by sudden fear, and vomiting bubbles. (Jacobson 2002: 261)

There are a number of other cases of these disorders in the literature, but rather than attempting a systematic translation into psychiatric terminology, which would take me well beyond my personal expertise, or discussing treatment in detail, I shall turn in the next section to look at a second body of material which contributes importantly to Tibetan understandings of *rlung*: the role of *rlung* in Buddhist Tantra.

## *Rlung* in the Context of Tibetan Buddhist Tantra (Vajrayāna)

So far, I have been discussing what disordered or unbalanced *rlung* does as a pathogenic factor in illness. When we move to *rlung* in the Tantric context, we come up against a different set of concepts, but one that intersects in a variety of ways with the ‘medical’ version of *rlung* as presented in the *Rgyud bzhi* and subsequent Tibetan medical literature.

*Rlung*, as I have already mentioned, flows through the body. We have already met the idea of five principal forms of *rlung* with specific functions in the body, and this scheme recurs in the Tantric context. Tantric theory and practice is, however, more concerned with transforming the flow of *rlung* as a spiritual technique. In the Tantric context, *rlung* is closely associated with mind or consciousness; the mind ‘rides’ on *rlung* like a rider on a horse, and both controls and is limited by this relationship. At more subtle levels, *rlung* and mind become more or less identical, two sides of the same coin (cf. e.g. Thurman 1994: 35–41; Ozawa-de Silva and Ozawa-de Silva 2011; Loizzo 2016).

In Tantric Buddhism, *rlung* flows through a structure of ‘channels’—*rtsa*, translating Sanskrit *nāḍī*—which meet at a sequence of focal points along the central axis of the body. These *’khor lo*, literally ‘wheels,’ in Tibetan, correspond to the *cakra* or ‘chakras’ that are familiar from Western New Age thought. There are numerous channels, but the three main ones are the central channel (Tibetan *dbu ma*), situated in the centre of the body, roughly parallel to the spinal column for much of its length, and two other channels that wind around this central channel, meeting with it at the *’khor lo,* where in the ordinary—unenlightened or unawakened—human being there are typically ‘knots’ or obstructions that block the free flow of *rlung* within the channel.

The various Tibetan Tantric traditions differ in detail in relation both to the practice and its philosophical interpretation (cf. Rinbochay and Hopkins 1979: 31; Cozort 1986: 44–45; Hartzell 1997: 606; Guenther 1992: 139–140; Dalai Lama 1982: 129–132; Yangönpa 2015: 45–59; Loizzo 2016; Marek and Oliphant 2017: 423). Thus, for example, there are disagreements about the exact form of the channels and disagreements concerning whether the distribution of the channels differs in men and women. There are differences about how the five ‘root winds,’ which we saw earlier in their medical context, relate to the process of achieving Buddhahood (e.g. Yangönpa 2015: 69–72). There are also disagreements about the degree to which the *rlung* and the channels have a material existence, particularly at the coarse level of ordinary perception. Tibetan doctors in particular—and many Tibetan Tantric practitioners and scholars were also trained in medicine—were well aware that the channels and *cakra* could not be identified in any straightforward way in the physical body. Here the *Rgyud bzhi*’s own account of the channels, which is famously difficult to interpret, and seems to include both physical and ‘subtle’ or immaterial channels, did not help matters much (J. Gyatso 2015: 193–249; see also Garrett and Adams 2008).

What is important though is that the flow of *rlung* through the channels is directly associated both with the process of rebirth and with the attainment of Buddhahood (awakening or Enlightenment), which within the Buddhist scheme of things is the contrary of rebirth. This is often described in terms of a contrast between ordinary, everyday ‘karmic *rlung*’ and the ‘wisdom *rlung*’ that leads to Buddhahood. Karmic *rlung* brings about rebirth and drives the ongoing process of *saṃsāric* life. As Elio Guarisco puts it, the karmic wind (i.e. *rlung*) is inseparable from thoughts and emotions:

The karmic wind is the mount for the eighty natural concepts (*rang bzhin brgyad cu rtog pa*), including attachment, anger, ignorance, and so on. These conceptions manifest during the life of the individual, and are like a pattern or cage in which the individual is trapped. (Guarisco in Yangönpa 2015: 73)

This karmic wind and the associated conceptions subside at the time of death, which is why death provides an opportunity for the attainment of Buddhahood. Yogic practitioners, however, aim to bind the karmic wind through their practice and thus gain freedom while still alive. This involves cultivating the ‘wisdom wind’:

While karmic wind represents the mind, wisdom wind represents the mind’s nature, the reality in which the subject-object duality is absent and objectifying thoughts cease. The mind’s nature is characterised by bliss, clarity, and nonthought. (Guarisco in Yangönpa 2015: 73)

Later in the same chapter, Guarisco quotes the late Dilgo Khyentse Rinpoche, widely recognised as one of the great Tantric masters of the twentieth century:

In fact, the wisdom wind and the karmic wind are the same thing. If this wind is brought under control, it engenders wisdom; if it is not controlled, it gives rise to the ordinary mind, together with its poisons. Thus the most important thing, at the perfection stage [of Tantric practice], is to work effectively on the wind… If, as a result, one attains mastery of the essence-drop [*thig le*, Sanskrit *bindu*], the mind, which is supported by it, will also cease to move, thereby giving rise to the experiences of bliss, clarity, and nonthought. (Cited by Guarisco in Yangönpa 2015: 78)

The essence drop or *thig le* here is a specific component of the flow of *rlung*, closely associated with both *bodhicitta*, the central motivational energy for the attainment of Buddhahood, and the physiological and psychological processes aroused during sexual intercourse, which are also circumstantial causes for the process of rebirth.^8^ In experiential terms, ‘mastery of the essence-drop’ involves freeing up through internal yogic practice the knots in the channels and accumulating the *thig le* (which supports or constitutes *bodhicitta*) in the central channel.

An important role is played in this process by the two other main channels, one on either side of the central channel. These two channels are also found in Śaivite yoga, in the Hindu tradition and the Vajrayāna yoga of Buddhist tantra. In Hindu tradition, they are called *iḍā* and *piṅgala*,^9^ and flow around the central channel, *suṣumnā*. *Iḍā* and *piṅgala* are associated with male and female, and with the subtle aspects of their respective sexual substances, semen and (menstrual) blood. The central channel is associated with the transcendence of the male–female and other dualities. The Buddhist equivalents of *iḍā, piṅgala* and *suṣumnā* are called *lalanā, rasanā* and *avadhūtī* in Sanskrit (*rkyang ma*, *ro ma* and *dbu ma* in Tibetan). They too correspond to male and female, semen and blood, white and red colours, and a series of other dualities, of which the principal is Wisdom and Means (*shes rab* and *thabs*).

Wisdom and Means (*prajñā* and *upāya* in Sanskrit) are central and complementary terms in Mahāyāna Buddhism They are two fundamental aspects of the path to Buddhahood. ‘Wisdom,’ also translated as discriminating awareness or insight, is the state of awareness that can cut through the misleading appearances of the everyday world. ‘Means’ is the method or technique by which that insight or awareness is attained. ‘Means’ is intrinsically linked to the compassionate motivation (*bodhicitta*) which brings about Buddhahood. Wisdom could be seen as passive and receptive, Means as active and outward-directed, which is why they are associated with female and male, but they are two sides of the same process, the goal of which is their transcendence in Buddhahood (Table 4). I shall return to this relationship later, since it is important for the relationship I shall be drawing in the next section between the Tantric subtle anatomy and Western understandings of the autonomic nervous system.

**Table 4:** Three principal channels

Sanskrit names (Hindu)	Sanskrit names (Buddhist)	Tibetan names	Associations	More associations
*iḍā*	*lalanā*	*rkyang ma*	red, solar	Means, Compassion
*piṅgala*	*rasanā*	*ro ma*	white, lunar	Wisdom, Insight
*suṣumnā*	*avadhūtī*	*dbu ma* (also *kun ’dar ma*)	beyond duality	Buddhahood

Much more could be said on the role of *rlung* and the subtle body in the Vajrayāna Buddhism. The above though should be enough to indicate the key importance of *rlung* and of the channels for Tantric enlightenment. A correlate to this is the importance of a healthy body, which is assisted through the practice of yogic exercises (*’phrul ’khor,* sometimes rendered in English as ‘yantra yoga’), which incorporate both physical movements and inner work with the breath and inner flows (Loseries-Leick 1997; Chaoul 2006).

Serious practice of this kind is done in prolonged retreat, ideally in its early stages under the close supervision of a lama. In modern times this is typically over a retreat period of 3 years and three fortnights (Kongtrul 1994). It is not surprising that such a heavy and intensive practice regime can go wrong. Meditation-caused illness is a familiar concept in Tibetan Tantric circles as in East Asia (cf Ahn 2017) and in Tibet is normally associated with *rlung* imbalance. There are textual resources and instructions on how to deal with these and other illnesses, which tend to focus on *rlung* practices and other meditational exercises (e.g. Lipman 2010; Marek and Oliphant 2017). Lamas may also respond to a problem of this kind by instructing the student to stop meditating, eat meat and other solid, grounding food and take part in ordinary everyday activities with friends in a relaxed environment, following the basic prescriptions for *rlung* illness given in the *Rgyud bzhi* and other Tibetan medical texts.

## The Autonomic Nervous System and Predictive Processing

What I have aimed to do in my article so far is to give a general picture of how *rlung* disorders are understood in Tibetan culture. To summarise, *rlung* disorders are associated with incorrect and unbalanced flows of *rlung*—a subtle, semi-material fluid-like substance associated with thought and emotion—within the body. They form a category within Tibetan medicine, but are also closely linked to Tibetan Tantric practice, since Tantra works directly with the *rlung* flows as part of a central body of techniques for attaining Buddhahood. Tantric theory also provides an account of what could be called optimal health, in terms of the proper flow of *rlung* leading to the minimisation of ‘karmic’ winds and ‘destructive emotions,’ and ultimately the achievement of Buddhahood. There is a substantial body of ideas within Tibetan culture, in both medical and Tantric scholarship, and among lay people, concerned with these matters.

In this section I turn to discuss—necessarily more briefly—Western ideas about the autonomic nervous system, along with developments in the theory of the emotions and the neuroscientific study of perception. This and the following section will provide the basis for a comparison between these Western ideas and the Tibetan model of *rlung* in the final section.

The study of the autonomic nervous system or ANS goes back some way in Western medical history, though the term itself dates from the work of John Newport Langley in the late nineteenth century (Oakes et al. 2016). Langley was also responsible for emphasising the complementary or antagonistic functions of the newly identified sympathetic and parasympathetic components of the system. Further important contributions were made by a series of scholars in the 1920s to 1940s, including Walter Bradford Cannon, Hans Selye and Walter Rudolf Hess. This led to a body of knowledge about patterns of activation of the ANS, and the role played within this by the sympathetic and parasympathetic components. In general terms, the sympathetic component is associated with the ergotropic (‘fight or flight’) response originally identified by Cannon, the parasympathetic with the trophotropic (‘relaxation’) response. While much of this was based on animal research, it was readily extended to human beings. Selye in particular was responsible for a general theory of stress that increasingly became applied to human beings as well as other animals, and which assimilated psychological stress and physical stress into a single mechanism (Samuel 2017). Our modern idea of ‘stress’ largely results from Selye’s work, and part of the package here is the idea that too much stress can damage the organism.

Meanwhile, the idea that the specific pattern of activation of the ANS was critical for the human organism as a whole, and that some forms of psychiatric illness might be linked to disorders of the ANS, also progressively developed. Ernst Gellhorn developed the idea of an optimal ‘tuning’ of the ANS, involving a specific balance of ergotropic and trophotropic responses, in the 1960s (Gellhorn 1967, 1970). Carroll Izard’s 1972 book *Patterns of Emotion* argued that phenomena such as anxiety and depression can be seen as combinations or patterns of a small number of fundamental emotions, each of which involves a specific neural substrate, a characteristic neuromuscular-expressive pattern and a distinct subjective or phenomenological quality (Izard 1972: 2). This led to a neurophysiological approach to psychiatric illness in which forms of depression or anxiety are associated with particular patterns of activation of the ANS (e.g. 1972: 176–178).

More recently, the work of Stephen Porges has suggested that the parasympathetic system might be viewed not just as an alternative set of responses but rather as a mechanism which has evolved phylogenetically in order to modulate and manage the sympathetic nervous system’s ‘fight or flight’ response (Porges 2011: 133–166). The emotions, he suggested, could be seen as an ‘evolutionary by-product’ of this process by which the ANS is regulated, in which the vagus nerve plays a central part. If the sympathetic nervous system deals with mobilising the organism for action, the parasympathetic deals with social communication, self-soothing and calming and inhibits the ‘fight or flight’ response in situations where it is inappropriate. Porges went on to suggest that psychiatric disorders such as borderline personality disorder, autism or depression might be understood in terms of the inadequate functioning of the vagus nerve and so of the parasympathetic nervous system (e.g. Porges 2011: 217–237). Trauma challenges the polyvagal mechanism, and PTSD and other responses to trauma and abuse can also be understood in terms of the effects on the parasympathetic regulation of the ANS (Porges 2011: 238–254).

Porges’s work has some relationship to what was to become the main initial explanatory framework for understanding Buddhist meditation, that advanced by Herbert Benson (1976, 1981). Benson, initially studying the Hindu mantra technique of Transcendental Meditation (TM), saw it as one of a range of cultural techniques found in all or almost all human cultures to activate the parasympathetic nervous system and so bring about the ‘relaxation’ (or trophotropic) response (Benson 1976, 1981). However, Benson’s model differs from Porges, in that Benson works with a straightforward dichotomy of ergotropic and trophotropic responses, involving the sympathetic and parasympathetic ‘system,’ respectively, while Porges sees the parasympathetic system as modulating and where appropriate inhibiting the sympathetic system. They also differ in that meditation is a conscious and deliberate activity, whether or not consciously undertaken to bring about a ‘relaxation response,’ while Porges is talking about a mode of functioning which may be acquired in the course of human socialisation but is not necessarily conscious.

Porges is only one of a variety of researchers in recent years who have suggested that psychiatric disorders might be linked to the functioning of the ANS. Neuroscientific explanations of autism spectrum disorders have received particular attention and have been linked to problems with interoception and sensory processing (e.g. Quattrocki and Friston 2014; Järvinen et al. 2015; DuBois et al. 2016). This goes along with a developing trend in neuroscience, often known as ‘predictive processing,’ which sees perception and the sensory input of the organism as a more complex matter than had previously been generally recognised.

The idea that perception involves interpretation of the incoming data is an old one, associated among others with the nineteenth-century physician and physicist Hermann von Helmholtz. Predictive processing takes this a step further, arguing that the neural system learns over time how to interpret sensory data more effectively, and in this way learns to make better predictions about how to live and operate in the world (Friston 2010; Hohwy 2013; Clark 2013, 2016). This learning is at more than a simple stimulus–response level, since it involves the way in which combinations of sensory data are interpreted. In this sense, the predictive processing model harks back to the anthropologist Gregory Bateson’s distinction between ‘Learning 1’ and ‘Learning 2’ in the 1960s (Bateson 1973). Much of the interest on the neuroscientific side is on how neurons might work to do this (e.g. Kanai et al. 2015). These neurons are not necessarily just in the brain, and the sensory input includes interoception regarding the internal state of the body as well as data about the ‘outside’ world. Thus, it necessarily involves the neural system as a whole (e.g. Barrett and Simmons 2015). For authors of a more philosophical orientation, such as Andy Clark, predictive processing also links up with the philosophical movement known as ‘enactivism’ and the associated idea of the ‘embodied mind’ (Maturana and Varela 1980; Varela, Thompson and Rosch 1991; Thompson 2010), a tradition which also derives in part from Bateson’s work in the 1960s. This tradition of thought sees our (conscious and/or unconscious) understanding of the world as constructed through our interaction with our environment, including our own bodies. Predictive processing implies that the neural system can change and transform so as to provide more adequate and effective functioning within the world. It also provides scope for this process to fail in some way, again offering a mechanism for understanding psychiatric disorders grounded in the operation of the autonomic nervous system (e.g. Quattrocki and Friston 2014), and the endocrine system, which is of course closely related to the ANS (Janušonis 2014).

## Emotion, Consciousness and Meditation

Before turning to look directly at the relationship between these Western ideas and the Tibetan model of the subtle body and the flows of *rlung*, I return briefly to the question of the role of the emotions within models such as those of Izard and Porges. The Western understanding of emotion is itself a complex area. The term itself is not very old, taking over in the nineteenth century from a series of earlier terms and categories (appetites, passions, sentiments, affections), and progressively developing a sense of opposition to rationality, intellect and conscious purpose:

The category of emotions, conceived as a set of morally disengaged, bodily, non-cognitive and involuntary feelings, is a recent invention. Prior to the creation of the emotions as an over-arching category, more subtlety had been possible on these questions. The ‘affections’, and the ‘moral sentiments’, for example, could be understood as both rational and voluntary movements of the soul, while still being subjectively warm and lively psychological states. It is not the case that prior to the 1970s no one had realised that thinking, willing and feeling were (and should be) intertwined in one way or another. Almost everybody had realised this. (Dixon 2003: 3)

What was, however, true of much of the writing on the emotions from the late nineteenth century onwards was an assumption that emotion was largely driven by physiology rather than consciousness. This can be found in classic form, for example, in William James’s famous essay, ‘What is an emotion?’ first published in 1884 (cf Dixon 2003: 207), in which James argues that perception causes a bodily reaction, and that our feeling of the bodily reaction constitutes the emotion. The precise relationship between physiological reaction, perception, cognition and emotion, and the relative emphasis placed on each, varies between scholars working on emotion, and there is no clear definition of what the term refers to. In addition, while the idea of a small number of fundamental emotional states, with distinct physiological bases, has been quite popular (Izard for example supported this approach), there is no consensus on whether this is a realistic model, or what these fundamental states might be.

That emotion is closely linked to the functioning of the ANS nevertheless became widely accepted. If, as Dixon suggests, most serious scholars accepted all along that ‘thinking, willing and feeling were… intertwined’ (Dixon 2003: 3), a natural move from here was to see rationality and the intellect as being *structured* by the emotions, seen as patternings of the ANS. The human organism might react differently, we might think and behave differently, depending on our current emotional state (i.e. pattern of activation of the ANS).

This model bears a perhaps significant resemblance to the mutual relationship between wisdom and means in the structure of the subtle body. In fact, the *cakra* of the subtle body have often been used, both in Asian and Western contexts, to represent a series of different sets of human responses, ranging from the physiologically driven, in the lower *cakra*, to the more subtle and socially oriented, in the higher ones. Normal activity in the everyday world of *saṃsāra* involves the outer channels, and flows of *prāṇa* (*rlung*) through the entire subtle body system. It is only when flows become ‘unbalanced,’ through an excess of *rlung* in particular areas, that problems manifest that might be understood in Western terms as psychiatric illness. The ultimate goal, however, at least in the Tibetan context, involves moving the *rlung* in from the outer channels, which represent various ways of becoming stuck in unproductive responses (the flow of the karmic winds) to focus on the wisdom wind, ultimately concentrated at the heart centre. The heart centre is linked to compassion, and the concentrating of *rlung* at the heart centre is also thought of as building up the compassionate motivation of *bodhicitta*, the fundamental motive cause for the attainment of Buddhahood.

Thus, attaining Buddhahood involves not only ‘balancing’ the emotional flows in the subtle body, but also attaining high levels of awareness of oneself and the external world, which are in any case not separate for an awakened Buddha. Healing psychiatric disorders, at least those which are understood in terms of ‘unbalanced flows’ in the subtle body, thus is integrated into a model whose eventual goal is the attainment of Buddhahood, which from this point of view could be seen as representing a state of perfect health, emotional balance, awareness and responsiveness.

A series of recent studies, criticising the early Western focus on meditation as primarily about the ‘relaxation response’ (Benson 1976, 1981), have argued that the cultivation of high levels of awareness is central to many forms of Buddhist meditation (Lutz et al. 2007, 2008; Britton et al. 2014; Amihai and Kozhevnikov 2014, 2015; Lumma et al. 2015). Some of these studies pointed to elements of attention and focus that appeared to be central to the understanding of meditation in Buddhism (Lutz et al. 2007, 2008), while others suggested that relaxation was not necessarily antithetical to awareness (Britton et al. 2014). Maria Kozhevnikov’s work, particularly her systematic comparison between two forms of Theravadin meditation and two forms of Tibetan meditation reported on in the 2014 and 2015 articles, demonstrated experimentally that much meditation clearly did involve states of arousal involving activation of the sympathetic nervous system (Amihai and Kozhevnikov 2014, 2015). It seems likely that Tantric meditation practice, both Buddhist and Hindu, can best be understood as providing access to states of high awareness and high functioning that differ from either ergotropic or trophotropic arousal in their basic forms (Kozhevnikov, this issue; see also Lidke 2016; Ruff 2016).

## Relating Tibetan and Western Approaches

Lidke’s 2016 article, looking at Śaiva tantric material, specifically the work of the great tenth century Kaśmiri Tantric philosopher Abhinavagupta, built an explicit connection between Tantric practice and the neurophysiology of the ANS, arguing that Tantric *sādhanā* in the Śaiva tradition can be understood as a form of training of the autonomic nervous system. Specifically, this training, through the outward and inward meditative gazes of his article’s title, involves a balancing of sympathetic and parasympathetic nervous systems, in order to ‘harness the potential,’ in Lidke’s words, ‘that comes from a simultaneous activation of these deeply integrated neurological systems’ (Lidke 2016: 5). Lidke’s Śaiva-based argument can readily be extended to Vajrayāna (Buddhist Tantric) meditation, and specifically to the so-called subtle body practices that I have discussed in earlier sections of this paper (Ruff 2016). The equivalent in Vajrayāna Buddhism of the inward and outward gazes of Abhinavagupta’s Śaiva tantra practice can be found both in the structured and symmetrical imagery of Tantric deities and Tantric *maṇḍalas* found in the generation stage of Anuttarayoga Tantric practice, and especially in the so-called completion stage processes, which involve the balancing of the two outer channels that wind around the central channel of the subtle body, and the gradual bringing of subtle fluid into the central channel.

The idea that these practices, involving the *cakra* and channels within the subtle body, through which subtle substances flow, can be seen as a kind of training of the autonomic nervous system has been around for a while, and this is where the two sides of the story I have been telling, the Tibetan and the biomedical, start to fit together. I referred to this idea myself in an article published 1989 on the Body in Buddhist and Hindu Tantra (Samuel 1989) and again in a book from 4 years later (Samuel 1993). I discussed it again in a recent edited volume on the subtle body in Asian and Western cultures (Samuel and Johnston 2013). As I pointed out then, gaining control over aspects of the autonomic nervous system also involves control over aspects of the endocrine system, which is quite literally a matter of internal flows. Here we can begin to see how traditional Tibetan or Śaivite models, on the one hand, and contemporary scientific understandings, on the other, might be expressed in different conceptual languages, but nevertheless have some degree of mutual compatibility.

This is not to say that the Tibetan accounts of the subtle body are to be taken as literal physiological descriptions, but I think it is possible to steer a middle way between an unqualified acceptance of either Western science in its present form or traditional Asian knowledge—which in fact was not as fixed as is often supposed (cf. Samuel 2013a). In particular, visualising and learning to operate with the subtle body can be seen, for the Tibetans, as a constructive process, intended to bring about an inner transformation, rather than as simply descriptive. Forms of meditation practice that involve repeatedly imagining the subtle body in a particular way—seeing the outer channels with their karmic winds, for example, as flowing into the central channel’s wisdom wind, can be seen as a kind of tuning of the nervous system enabling a higher level of performance, a conscious mechanism for improving predictive coding.^10^

If one can see a convergence of Tibet and Western ideas here, in terms of the achievement of high levels of performance, health and balance, one can also see a similar convergence in terms of ideas of states of imbalance and of disorders. As I noted earlier, there are long-standing suggestions that pathologies of the functioning of the nervous system might underlie psychiatric illness. There is at least a case for similarities and parallels at several levels, including the Tibetan role of the root *kleśas* or destructive emotions in the genesis of illness.

Here then is a potential area of compatibility between Tibetan and Western modes of scholarly and scientific understanding, both at the level of practices and at the level of technique. However, before assuming a genuine relationship between the two sides, it is worth spelling out some of the similarities and differences between the two frames of explanation.

To begin with similarities: one can certainly make a parallel of sorts between the two aspects of the autonomic nervous system (sympathetic and parasympathetic) and the two outer channels of the subtle body practice. Lidke’s argument, as extended by Ruff, sees the Tantric meditational practices as somehow integrating, transcending, the dualism of the two channels, through a kind of ‘re-tuning’ of the two aspects of the autonomic nervous system to enable higher and more integrated levels of functioning.

There is also at least a general parallel between the specific content of the two systems. The sympathetic system is active, involved in outward movement, in engagement with the external world, while the parasympathetic system is inward looking, involved in withdrawal from action and engagement. The two outer Tantric channels encode a similar opposition, between Means and Wisdom, the first active, externally engaged, outward looking, while the second is inward looking, involving insight rather than engagement.^11^

The way in which the Tibetan vocabulary sees emotion as a fluid or quasi-fluid substance that moves around the body is also both similar and different to how emotions tend to be thought about in Western cultures, where they are often seen as located within the body (e.g. the heart), but as static rather than moving.^12^ Yet it maps rather well onto the understanding of the emotions as linking to actual flows in the endocrine system which are associated with, for example, the ergotropic and trophotropic responses (e.g. cortisol and adrenaline for the ergotropic response). The relationship between endocrine system and autonomic nervous system also has an intriguing parallel to that in Tibetan thought between *rlung* and consciousness, in which consciousness both ‘rides’ on *rlung* but is also more or less indissoluble from it, though I do not think I would want to push this too far.

There are also important differences between the two systems, and if we are to build on the connections and correspondences, these differences need our serious attention. One is that the Western model operates in terms of explicitly physiological variables. It may be assumed or supposed that consciousness is associated with the electrical functioning of the autonomic nervous system and the hormonal activity of the endocrine system, but the Western explanatory model is generally used in a way that sidesteps the existence of consciousness. Much of the experimental work behind the Western model was carried out with animals, so that the consciousness of the experimental subjects was generally inaccessible even had it been thought appropriate to include it. There are of course Western modes of thought which work in terms of consciousness, including some in the field of psychotherapy, but they are generally seen as incompatible with the kind of experimentally driven, empiricist model underlying most neuroscience. We might consider how incompatible they really are, and whether it might be possible to build some bridges in this area. There have been interesting recent attempts, for example, to build more precision into modes of first-person centred phenomenological enquiry (cf Sheehy, 2017).

In any case the Tibetan mode of thought operates somewhere in between. The unity-in-duality of consciousness and *rlung* within the subtle body is posed at a level somewhere *between* mind and body, neither purely material nor purely at the level of consciousness.

Another point of difference is that the Tibetan mode implies the presence of karma as a causal force behind the operation of the flows in the subtle body. The karmic wind is the ongoing continuity (*rgyud*) of the individual, transmitted from one rebirth to the next. ‘If this wind is brought under control, it engenders wisdom; if it is not controlled, it gives rise to the ordinary mind, together with its poisons.’ (Dilgo Khyentse Rinpoche, cited by Guarisco in Yangönpa 2015: 78). Can we make any sense of this, without being caught up in a whole set of essentially unverifiable assumptions from Buddhist philosophy? Of course, it is also possible to turn this question around into a query about the equally unverifiable background assumptions behind Western science, and their consequences for the society in which we live. Yet this has often proved a sticking point in the dialogue between Western and Tibetan thought (e.g. Samuel 2014).

A further issue bears on the problematic and tense relationship between the various modes of discourse of natural science, social science and humanities which often invests discussion in areas such as this, at a level a little below explicit consciousness. The scientific mode tends to assume that it is dealing with empirical reality. Western scholars approaching concepts such as those of *rlung* are likely to treat them rather as metaphorical, as alluding to a reality through visual, verbal or tactile imagery rather than to describe it in some direct way. When we speak of adrenalin being produced within the endocrine system as part of the ergotropic response, this has the nature of a statement about empirical reality. When we speak of mind riding on the currents of *rlung* within the subtle body, we are more likely to think of this as a metaphorical statement.

Yet the contrast between the two statements is not as straightforward or as absolute as this might suggest. Natural science too can be seen as having an underlying metaphorical component, in the structure of background assumptions, the framework of ideas within which its hypotheses are constructed (Samuel 1990). These can perhaps be, and in some though not all fields of science typically are, described mathematically in ways that are not used in traditional Asian modes of thought, but this does not make them less arbitrary in the sense of going beyond anything deducible from empirical observation. Equally, the metaphorical structure of Tibetan thought about quantities such as *rlung* may not be entirely free-floating away from any kind of basis in reality, whether that reality be the reality of physiology or that of consciousness. Yet how do quantities such as *rlung* relate to an organic basis?

Here I think it is useful to return to the suggestion above that *rlung* can be seen less as a fixed description of human neurophysiology and more as a way to visualise and grasp it, and so to operate with it. We can begin to see how this might operate through the work of Lifshitz and others (Lifshitz 2017) on the role of suggestion in meditation practices. I spoke above of how this might help us to understand the ‘re-tuning’ of the ANS through Tantric meditation to achieve higher levels of performance. It is worth noting that this ‘re-tuning’ also contains an intrinsic moral and ethical component. The practitioner is visualising his or her body–mind complex not just as a mechanism that may be coaxed into operating more efficiently for the purposes of everyday life, but as a vehicle for the attainment of Buddhahood in order to relieve the sufferings of other living beings. The language of karmic and wisdom wind, of substances flowing through the mind–body which are homologised with *bodhicitta*, the central motive force of Buddhahood, and focussed in the central channel precisely to aid the achievement of Buddhahood, and the like, means that one cannot do these practices correctly without being at the same time drawn into the transformative project of the Buddhist teachings.

That brings to mind that the Tibetan model of psychiatric disorders differs from the Western model in that its goal is not simply the curing of the disorder, so that the patient can return to ‘normal’ life, but an active state of health which implies positive social engagement with the welfare of others.^13^ The point is to become a better person, and if the disease is cured, this is in a way a side-effect. Put otherwise, like any of the sufferings of *saṃsāric* life, psychiatric illness offers an opportunity for transcendence. In Norbu’s account of Ugyen Tendzin’s experience of *snying rlung*, Adzom Drugpa’s ritual therapy for the *snying rlung* was followed by taking on Ugyen as a disciple, who was eventually to reach the highest levels of Buddhist yogic attainment.

Buddhahood may seem a distant goal in contemporary Western culture, for all of the currently fashionable status of Buddhist-derived practices. Perhaps, though, one area in which Tibetan models have something important to contribute to our evolving understanding of psychiatric disorders is to remind us that the psychiatrist remains a healer of the soul, and that healing the soul involves leading the patient towards a fuller participation in humanity’s collective search for a better life, and a better world.

## Footnotes

1 The same is true of ‘blood’ (*khrag*) which will occur later in this article.

2 Cf. Goleman 2004, which has some interesting material on the contrast between Western and Tibetan modes of conceptualising ‘emotions’.

3 *Rgyud bzhi, Bshad rgyud*, chapter 8: khyad par rgyu ni ma rig las byung ba’i || ’dod chags zhe sdang gti mug dug gsum las || ’bras bur lung mkhris bad kan nyes pa skyed || (Rgyud bzhi, n.d., 51).

4 The name is a little misleading, because while written in the style of the Tantras, the *Rgyud bzhi* is a medical work, not a Tantric text.

5 *Rgyud bzhi*, *Man ngag rgyud*, chapter 2: bye brag dbye bas srog’dzin gyen gyu dang || khyab byed me mnyam thur sel rkyang ba lnga ||.

6 *Rgyud bzhi, Bshad rgyud*, chapter 5.

7 Jacobson, who interprets *srog rlung* in general as a combination of the DMS-IV categories of Generalized Anxiety Disorder and Major Depression, describes six cases in detail in his PhD (Jacobson 2000); Kalsang (a pseudonym), one of these, is also discussed at length in Jacobson 2002 (see also Jacobson 2007). He also provides some information about treatment. Janes gives further examples of *rlung* disorders in his articles (e.g. Janes 1999). Another interesting case study of *khrag rlung* is given by Audrey Prost (Prost 2008: 93–96). Benedict et al 2009 looks at *srog rlung* in relation to PTSD in the context of traumatised Tibetan refugee monks, pointing out some of the differences from DSM categories (Benedict et al 2009). In a recent article, Holečko discusses the treatment of *snying rlung* (Holečko 2015).

8 On the complex relationship between *thig le* and *bodhicitta*, see also Wangchuk 2007, especially 217–225.

9 Hartzell suggests that the names in Hindu tradition for the two outer channels, *iḍā* and *piṅgala*, may derive ultimately from Vedic terminology (1987: 114–117).

10 Michael Lifshitz noted in a comment on a presentation of this material at McGill that there is extensive evidence for the role of suggestion in meditation experience, going back to early work on Transcendental Meditation. He has surveyed much of this work in his recent PhD thesis (Lifshitz 2017). He suggested in subsequent conversations that work on predictive processing (see above) provides both further support for such a self-reinforcing loop mechanism behind subtle body processes, and models for how this might work in neurophysiological terms. Lifshitz, Laurence Kirmayer, and myself hope to develop these suggestions further in a forthcoming paper.

11 The Hindu terminology is parallel though somewhat different.

12 Cf. the often-cited work of Lakoff and Johnson (1980), though they tend to see their scheme as more cross-culturally uniform than I think can be justified.

13 Similarly, one cannot do practices such as the long-life practices I have discussed in other writings without being caught up in the project of Buddhahood (Samuel 2013b, 2016).
